# Influence of HTLV-1 on the clinical, microbiologic and immunologic presentation of tuberculosis

**DOI:** 10.1186/1471-2334-12-199

**Published:** 2012-08-28

**Authors:** Maria de Lourdes Bastos, Silvane B Santos, Anselmo Souza, Brooke Finkmoore, Ohana Bispo, Tasso Barreto, Ingrid Cardoso, Iana Bispo, Flávia Bastos, Daniele Pereira, Lee Riley, Edgar M Carvalho

**Affiliations:** 1 Serviço de Imunologia do Complexo Hospitalar Universitário Professor Edgard Santos, Universidade Federal da Bahia, Salvador, BA, Brazil; 2 Escola Bahiana de Medicina e Saúde Pública, Salvador, BA, Brazil; 3 Hospital Especializado Octávio Mangabeira, Salvador, BA, Brazil; 4 University of California at Berkeley, Berkeley, USA; 5 Instituto Nacional de Ciência e Tecnologia de Doenças Tropicais (CNPq/MCT), Salvador, BA, Brazil; 6 Serviço de Imunologia, 5 andar, Hospital Universitário Professor Edgard Santos, Rua João das Botas s/n, Canela, Salvador, BA, 40110-160, Brazil

**Keywords:** HTLV-1, Tuberculosis, Mycobacterium tuberculosis

## Abstract

**Background:**

HTLV-1 is associated with increased susceptibility to *Mycobacterium tuberculosis* infection and severity of tuberculosis. Although previous studies have shown that HTLV-1 infected individuals have a low frequency of positive tuberculin skin test (TST) and decreasing in lymphoproliferative responses compared to HTLV-1 uninfected persons, these studies were not performed in individuals with history of tuberculosis or evidence of *M. tuberculosis* infection. Therefore the reasons why HTLV-1 infection increases susceptibility to infection and severity of tuberculosis are not understood.The aim of this study was to evaluate how HTLV-1 may influence the clinical, bacteriologic and immunologic presentation of tuberculosis.

**Methods:**

The study prospectively enrolled and followed 13 new cases of tuberculosis associated with HTLV-1 (cases) and 25 patients with tuberculosis without HTLV-1 infection (controls). Clinical findings, bacterial load in the sputum, x-rays, immunological response and death were compared in the two groups.

**Results:**

There were no differences in the demographic, clinical and TST response between the two study groups. IFN-γ and TNF-α production was higher in unstimulated cultures of mononuclear cells of case than in control patients (p < 0.01). While there was no difference in IFN-γ production in PPD stimulated cultures, TNF-α levels were lower in cases than in controls (p = 0.01). There was no difference in the bacterial load among the groups but sputum smear microscopy results became negative faster in cases than in controls. Death only occurred in two co-infected patients.

**Conclusion:**

While the increased susceptibility for tuberculosis infection in HTLV-1 infected subjects may be related to impairment in TNF-α production, the severity of tuberculosis in co-infected patients may be due to the enhancement of the Th1 inflammatory response, rather than in their decreased ability to control bacterial growth.

## Background

The human T cell lymphotropic virus type 1 (HTLV-1) has a worldwide distribution; it is most prevalent in Central and South America, Central Africa and southwestern Japan [1]. HTLV-1 infects predominantly CD4 T cells inducing cell activation and proliferation of both CD4 and CD8 T cells [2,3]. Moreover, the high production of pro-inflammatory cytokines (TNF-α and IFN-γ) has been associated with central nervous system (CNS) damage and the development of HTLV-1 associated myelopathy (HAM) [2,3].

There is evidence that HTLV-1 infection increases severity and susceptibility to strongyloidiasis, scabies and tuberculosis [4-9]. In patients co-infected with HTLV-1 and *Strongyloides stercoralis* the exaggerated Th1 immune response and increased regulatory T cell response decrease the Th2 immune response, which plays a pivotal role in host defense against helminthes [9-12]. HTLV-1 infected individuals have two to four-fold increased chance to acquire tuberculosis [6,13-15]. Additionally, in one retrospective study, it was observed that HTLV-1 increased the mortality rate of tuberculosis [8]. Since the frequency of responders to tuberculin skin test (TST) [16-18] and in vitro lymphocyte proliferation stimulated by protein purified derivate (PPD) is lower among HTLV-1 infected individuals with tuberculosis than in HTLV-1 uninfected persons [19], it has been proposed that a decrease in type 1 immune response to mycobacterial antigen may increase tuberculosis susceptibility and severity. In this study the clinical, radiologic, immunologic, and bacteriologic features of patients with tuberculosis and HTLV-1 infection were compared with those who had only tuberculosis infection.

## Results

There was no significant difference in the demographic characteristics (age, gender, ethnicity, monthly income and nutritional status) between the 13 patients with tuberculosis and HTLV-1 infection and 25 patients with only tuberculosis. A large proportion of case (54%) and control patients (64%) were underweight (body mass index < 18.4) and the use of alcohol was also similar in both groups. The clinical manifestations, response to TST and the presence and quantification of acid-fast bacilli (AFB) in the sputum in the two groups are shown on Table 1. The presence of fever, asthenia, anorexia and weight loss were similar in the two groups (p > 0.05). In both groups the frequency of hospitalization for tuberculosis, previous tuberculosis and treatment abandonment for tuberculosis was high. There was no difference regarding the frequency of responders to the TST as well as the size of induration. At the time of admission, two patients with HTLV-1 infection and tuberculosis had been receiving therapy for tuberculosis for 28 and 35 days, respectively; in both, the sputum test was negative. However, previous to the therapy, both were documented to have AFB in the sputum, which also grew *M. tuberculosis* in culture*.* They were admitted because of toxicity to anti-tuberculosis drugs. No difference in the bacillary load was observed in the two groups at admission. The drug sensibility test revealed one *M. tuberculosis* isolate with multidrug resistance and another with resistance to isoniazid and streptomycin among the cases. In the control group, there was one isolate with resistance to isoniazid, rifampicin and ethambutol and one with resistance to streptomycin. The findings of the chest x-rays were similar in the 2 groups. Cavitation was observed in more than 60% of the patients with or without HTLV-1 infection. Fibrosis and atelectasis occurred in about 50% of the cases and controls. Parenchymal destruction was higher (15.3%) in patients with HTLV-1 infection and tuberculosis than in patients with only tuberculosis. The radiologic findings in patients with HTLV-1 and tuberculosis were not compatible with those observed in patients with T cell impairment. The most common findings were fibrosis, atelectasis, pleural thickening and cavitation. All of them were similar in the two groups but the parenchymal destruction lesions were 3.5 fold higher in patients with HTLV-1 infection and tuberculosis than in patients only with tuberculosis.

**Table 1:** Clinical and microbiologic characteristics of tuberculosis patients with or without HTLV-1 infection

**Characteristic**	**TB with HTLV-1**	**TB without HTLV-1**	**p-value**
	**(n = 13)**	**(n = 25)**	
	**n (%)**	**n (%)**	
**Symptoms**			
Fever	11 (84.6)	21 (84.0)	0.67
Asthenia	13 (100)	25 (100)	-
Anorexia	11 (84.6)	21 (84.0)	0.35
Weight loss	11 (100)	22 (95.7)	-
Prior hospitalization for TB	6 (46.2)	7 (28.0)	0.26
Prior treatment for TB	7 (53.8)	12 (48.0)	0.21
History of treatment abandonment	6 (46.2)	7 (28.0)	0.45
**Clinical examinations**			
Helminth infections	2 (15.4)	3 (12)	0.63
PPD positive	10 (76.9)	14 (63.6)	0.71
Mean PPD (mm) among PPD positive tests, ± SD	12.9 ± 4.0	10.5 ± 4.8	0.23
**Sputum bacillary load**			
0	2 (15.4)^*^	0 (0)	
1+	5 (38.4)	11 (44.0)	
2+	2 (15.4)	3 (12.0)	
3+	4 (30.8)	11 (44.0)	

TB = tuberculosis; HTLV-1 = human lymphotropic virus type 1; SD = standard deviation.

The two patients had already been treated for tuberculosis for one month previous to the admission. Before therapy the smear microscopy result was positive.

The therapy for tuberculosis was similar in both groups. The majority of the patients received rifampicin, isoniazid and pyrazinamide. In 33.3% of the cases and in 28% of the controls, patients also received ethambutol. Alternative drugs were given to 15.4% of the cases and to 8% of controls.

**Figure 1:**
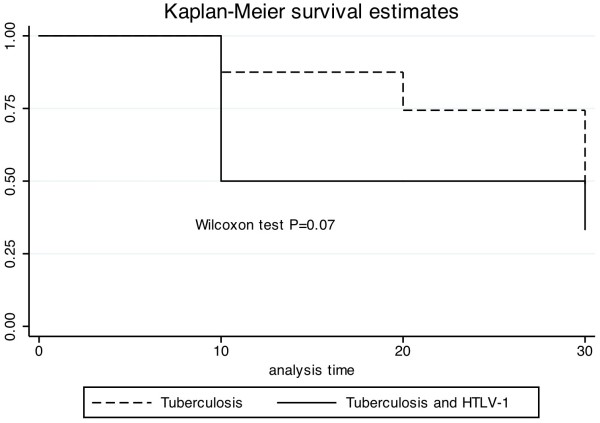
**Survival analysis of sputum smear conversion for acid-fast bacilli during tuberculosis treatment.** Tuberculosis patients with and without HTLV-1 infection, who remained test positive by acid-fast bacilli (AFB) smear microscopy after 10 and 20 days of tuberculosis treatment were evaluated with Kaplan-Meier survival estimates.

Figure 1 shows the survival analysis of patients whose sputum test became negative after 10 and 20 days of therapy. As previously stated, two patients with HTLV-1 infection and tuberculosis were already on therapy and the sputum test was negative. On day 10, while 50% of the patients with tuberculosis and HTLV-1 still had AFB in the sputum, almost 90% of patients with tuberculosis only remained eliminating bacilli. At day 20, 36.4% of the patients with HTLV-1 infection and tuberculosis and 75% of those with tuberculosis only were smear test positive for AFB (p = 0.07; Kaplan-Meier test). However, sputum test conversion occurred faster in patients with HTLV-1 infection and tuberculosis.

The main side effects observed were hepatotoxicity and gastric intolerance (nausea, abdominal pain and vomit) observed in, respectively, 38.5% and 23.1% in the cases and in 28% and 36% in the controls (p > 0.05). While death was observed in 2 (15.4%) of the patients with HTLV-1 infection and tuberculosis, none of the patients died in the group only with tuberculosis. Death occurred in one woman and one man. They both had cavitary lung disease, fibrosis, atelectasis and tissue destruction on chest x-rays. Death was due to acute respiratory insufficiency in one case and sepsis in the other.

Regarding HTLV-1 infection, the majority of the cases were HTLV-1 carriers. Four patients had overactive bladder that is considered to be an oligosymptomatic form of HAM. The proviral load was quite variable ranging from 8079 to 499742 copies/10^6^cells.

**Figure 2:**
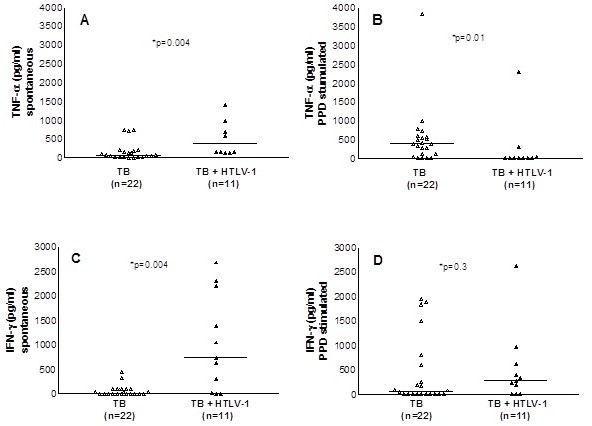
**TNF-α and IFN-γ production with or without stimulus with PPD.** TNF-α and IFN-γ production was determined by ELISA in supernatants of PBMC culture from co-infected patients and patients with tuberculosis only unstimulated (**A** and **C**) or stimulated with PPD (**B** and **D**). Results of PPD stimulated cultures represent values subtracted from the IFN-γ production in unstimulated cultures.

The spontaneous and PPD induced TNF-α and INF-γ production in supernatants of PBMC are shown in Figure 2. In cultures without stimulus, the median TNF-α level among the group with HTLV-1 infection and tuberculosis (368 pg/mL) ranging from 128 to 5527 pg/mL was higher (p = 0.004) than that observed in patients with only tuberculosis (73 pg/mL), which ranged from 0 to 738 pg/mL (Figure 2A). In cultures stimulated with PPD, the median and range of TNF-α level in the co-infected patients, 0 pg/mL (0 – 2295 pg/mL), was lower (p < 0.01) than in patients with only tuberculosis, which was 386 pg/mL (0 – 3847 pg/mL) (Figure 2B). The production of IFN-γ in unstimulated cultures in patients with HTLV-1 infection and tuberculosis 732 pg/mL (0 – 2677 pg/mL) was higher (p = 0.004) than in patients with only tuberculosis, which was 15 pg/mL (0 – 444 pg/mL) (Figure 2C). There was no difference between the groups in the production of IFN-γ in PPD stimulated cultures (Figure 2D). There was no difference (p > 0.05) in the production of IL-10 in both unstimulated and PPD stimulated cultures; majority of the co-infected patients (60%) and 45% of patients with tuberculosis without HTLV-1 infection had no detectable IL-10 production (data not shown). Addition of anti-IL-10 monoclonal antibody also did not enhance IFN-γ production (p > 0.05) in either group (data not shown).

**Figure 3:**
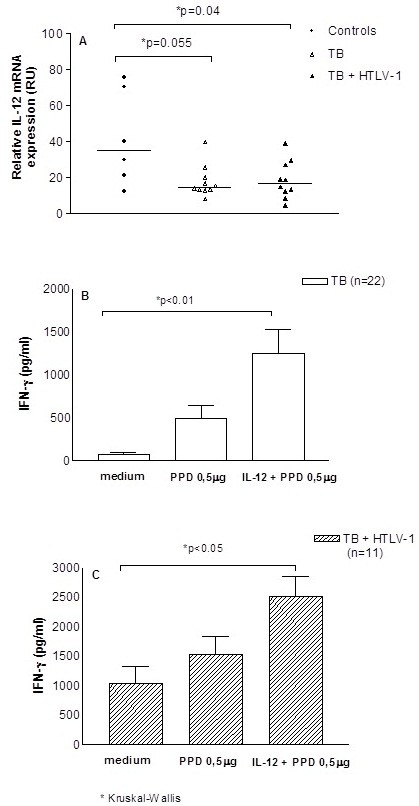
**Expression of mRNA for IL-12 and IFN-γ production after exogenous addition of PPD plus IL-12.** mRNA for IL-12 was determined by RT-PCR in unstimulated cultures of PBMC from seronegative healthy subjects, co-infected patients, and patients with tuberculosis only (A). IFN-γ production was evaluated by ELISA in supernatants of PBMC cultures unstimulated or stimulated with PPD and PPD plus IL-12 in the concentration of 50 μg/ml (B and C).

The expression of mRNA for IL-12 in unstimulated cultures and the production of IFN-γ in PBMC cultures stimulated with PPD and in cultures with PPD plus exogenous addition of IL-12 are shown in Figure 3. The expression of mRNA for IL-12 in unstimulated cultures was lower in tuberculosis patients with or without co-infection with HTLV-1 than in seronegative controls (Figure 3A). Exogenous addition of IL-12 in cultures stimulated with PPD enhanced IFN-γ production similarly in both groups (Figures 3, B and C).

## Discussion

The association between HTLV-1 infection and tuberculosis is widely documented, but little is known about the influence of HTLV-1 on the outcome of *M. tuberculosis* infection [6,13-15]. In the present study, the two cases of death occurred among co-infected patients, but no impairment in the T cell response to PPD was observed *in vivo* and in vitro*.* However, while there was decrease in TNF-α production in PPD stimulated cultures, the INF-γ production in unstimulated cultures was higher in co-infected patients than in patient only with tuberculosis. These data together with the observation that the sputum smear conversion was faster in the cases compared to controls, suggests that impairment in innate immune response may contribute to acquisition of tuberculosis, and that severity of the disease may be due to exaggerated inflammatory response observed in co-infected patients.

One important limitation of the present study was the characteristics of patients with tuberculosis. The admission for tuberculosis patients is usually due to severity of disease, severe adverse reactions to anti-tuberculosis drugs, history of treatment abandonment and documentation of multidrug-resistant *M. tuberculosis*[20]. In our case, in addition to the presence of one or more of these factors, a large percentage of the patients were underweight and malnourished, which may have also influenced the clinical course of tuberculosis. Therefore, a similar type of the study should be performed in out-patients with less severe tuberculosis manifestations to determine if the viral infection may change the symptoms and x-rays findings of tuberculosis. Nevertheless, since the frequency of these factors was similar in tuberculosis patients co-infected or not with HTLV-1, this study gives support to the previous observation that HTLV-1 infection worsens the course of tuberculosis [8].

While there is good evidence that HTLV-1 infection increases the risk for tuberculosis [6,13-15], very little is known about the clinical course of tuberculosis in such patients. In a retrospective study, it was shown that death was significantly more frequent in patients with tuberculosis and HTLV-1 infection than in patients who had only tuberculosis [8,14]. However no information regarding microbiological, radiological or clinical data were provided to determine how HTLV-1 can exacerbate the clinical course of tuberculosis. As previous studies have shown that subjects infected with HTLV-1 have a decreased immune response “*in vivo*” and “in vitro” to PPD [17-19,21], one possibility is that HTLV-1, by decreasing the immune response to *M. tuberculosis,* not only increases susceptibility but also makes tuberculosis more severe. The results presented here did not show any difference in the frequency of patients who respond to the TST as well as in the size of the induration when the two groups were compared. These data differ from the literature, but the population studied here was quite different from those included in previous studies. In the other studies, data were generated from HTLV-1 infected subjects without documented active tuberculosis or even without history of tuberculosis or vaccination with BCG [16-18]. Here all patients had active tuberculosis as documented microbiologically.

In vitro studies showed decrease in lymphocyte proliferation and secretion of cytokines upon stimulation with *M. tuberculosis* antigen in HTLV-1 infected subjects compared to controls [19,21]. Similar to the *in vivo* observations, the in vitro studies were also performed in HTLV-1 infected patients without history of tuberculosis. However, it is known that abnormalities in T cell or in antigen present cell function occur in HTLV-1 infection [21-23]. Therefore impairment in the Th1 type of immune response to mycobacterial antigens may increase susceptibility to tuberculosis. However, our clinical radiologic, microbiologic and immunologic data did not show any evidence of impairment in the T cell immune response to mycobacterial antigens in patients with tuberculosis and HTLV-1 infection. For instance patients with HIV infection have impairment in granuloma formation, atypical chest x-ray findings, and slower time to bacterial conversion [24]. Here all patients had AFB in the sputum. The majority had cavitation and fibrosis on chest x-ray, and parenchymal destruction was more frequent in patients with tuberculosis and HTLV-1 infection. These are findings observed in immunocompetent patients with tuberculosis. Moreover, there was no difference in the frequency and size of the TST and there was no difference in IFN-γ production in the two groups in PPD stimulated cultures. Others have found increase in bacillary load in patients with HTLV-1 infection and tuberculosis [14]. In this study we did not find any difference in the bacillary load, and the sputum test became negative faster in patients with tuberculosis co-infected with HTLV-1. Thus, the exaggerated inflammation with HTLV-1 infection may have contributed to the rapid sputum smear conversion and to the severity of tuberculosis. For instance mice lacking IL-27 receptors and mice infected with a *M. tuberculosis mce1* operon mutant cannot modulate immune response and mount an exaggerated inflammatory response associated with control of bacterial multiplication, but survival is decreased due to severe damage of the lung [25,26].

Compared to seronegative controls, expression of IL-12 was lower in both groups and exogenous addition of IL-12 enhanced IFN-γ production in a similar way in tuberculosis patients co-infected with HTLV-1 and in patients with tuberculosis. However, while there was no difference in IFN-γ production among the groups, in PPD stimulated cultures, TNF-α was lower in PPD stimulated cultures of patients co-infected with *M. tuberculosis* and HTLV-1. TNF-α plays a key role in protection against *M. tuberculosis*[27,28]. Actually emphasis has been given for the important role of innate immune response in the control of *M. tuberculosis* infection [29,30]. In such a case, the increased susceptibility of HTLV-1 infected individuals to *M. tuberculosis* may be due to impairment in TNF-α synthesis after exposure to *M. tuberculosis*, as well as in other component of the immune response not yet determined. However, as IFN-γ production is produced in high levels in HTLV-1 infection and a type 1 immune response to *M. tuberculosis* antigens is not suppressed, granuloma formation is observed and bacterial growth is controlled. Additionally, as HTLV-1 infection is associated with an exaggerated inflammatory response, there is more tissue damage, which causes severer tuberculosis in HTLV-1 infected subjects.

## Conclusions

While the increased susceptibility of HTLV-1 infected patients to acquire tuberculosis may be due to impaired TNF-α production, the severity of tuberculosis in HTLV-1 infected individuals may be related to an increased inflammatory response.

## Methods

The study participants included 13 patients with diagnosis of tuberculosis and HTLV-1 infection and 25 patients who had tuberculosis without HTLV-1. All patients with tuberculosis consecutively admitted to Hospital Especializado Octávio Mangabeira (HEOM), Salvador, Brazil, from August 2007 to July 2009 were tested for HTLV-1 by ELISA and those who tested positive underwent confirmation by a Western blot test. The inclusion criteria were age between 18 to 60 years, diagnosis of tuberculosis with documentation of acid-fast bacilli (AFB) by microscopy in the sputum and serologic test for HTLV-1. Exclusion criteria included pregnant women, patients with diabetes, kidney failure, HIV infection and use of immunosuppressive drugs. For each patient with tuberculosis and HTLV-1 infection, two controls with only tuberculosis matched by gender and age plus or minus five years of age were included. A case was defined as a patient with two sputum tests positive for AFB by microscopy and a positive ELISA test for HTLV-1 confirmed by Western blot. A standardized questionnaire soliciting demographic and clinical information was completed for each participant at the time of enrolment into the study. A pulmonary specialist (MLB) examined each patient and reviewed laboratory test and the x-ray results. The patients were evaluated at entrance into the study, and at 10, 20 and 30 days after admission. An informed consent was obtained from all participants and the study was approved by the Ethical Committee of the HEOM.

### Laboratory tests

The TST was performed by trained nursing staff at HEOM with 0.1 ml of PPD RT23 (2 tuberculin unit [TU], Statens Serum Institute, Copenhagen, Denmark). The reaction was read 72 h later. The cut-off point for a positive reaction was > 5 mm induration.

Serum samples were screened for antibodies to HTLV-1 by ELISA (Murex HTLV-1, Abbot, Dartford, UK) and confirmed by a Western blot analysis (HTLV Blot2.4, Genelabs, Singapore) performed in accordance with the manufaturer’s instructions. Samples were also screened for antibodies to HIV-1 by ELISA (Ortho-Clinical Diagnostics, Raritan, NJ, USA). Sputum of each patient was examined by the Ziehl-Neelsen staining method and the bacillary load was classified as 1+, 2+, 3+ or negative. Culture of the sputum was also performed in Lowenstein-Jensen culture media.

### Immunological studies

Peripheral blood mononuclear cells (PBMC) were obtained from heparinized venous blood by density gradient centrifugation using Ficoll-Hypaque (Sigma Chemical Co, St. Louis, MD, USA). After washing with saline, the cells were adjusted to 3x10^6^ml and cultured in RPMI 1640 (GIBCO Grand Island, NY, USA) plus 5% heat inactivated human AB serum supplemented with gentamicin and glutamine. Cells were incubated only with medium or stimulated with PPD at concentration of 1μg/ml for 72 hours at 37°C in 5% CO_2_. Determination of TNF-α, IFN-γ and IL-10 was performed by ELISA with monoclonal anti cytokine antibodies [3]. Data expressed in pg/ml represent the amount of protein detected in the supernatants of cultures. The mRNA for IL-12 was performed by real time PCR. HTLV-1 seronegative individuals were used as controls. RNA from cell lysates was isolated in Tri Reagent Solution (Ambion, Applied Biosystems, Foster City, CA, USA). The concentration and purity of RNA as well as DNA synthesis were assessed as previously described [31]. Briefly, for relative quantitative Real Time PCR cDNA specific primers for IL-12 and reference gene HPRT were purchased from RealTimePrimers.com (Real Time primers, LLC, 7304 Mountain Ave, Elkins Park, PA 19027, USA). Relative quantitative PCR reactions were performed on a Real-Time PCR system Step OnePLUS AB Applied Biosystems in a 96-well microtiter plate and a final volume of 10 μL using 0–5 μL of cDNA, 2–5 μL of 2X SYBR Green Master mix (AB Applied Biosystems, Foster City, CA, USA) and 30 pM specific primer mix. The cycling conditions were as follows: 10 min polymerase activation at 95°C followed by 50 cycles of 15 s at 95°C and 1 min at 60°C. All samples were amplified in duplicate, and two negative controls per primer pair were included in each run. Melting curve analysis was performed immediately after amplifications. HPRT mRNA expression was used for normalization. Relative expression levels were obtained as mean ΔCT for each gene using the software Step OnePLUS^TM^ Software v2.0 (AB Applied Biosystems). Data for stimulated cultures represent the production of cytokine obtained in culture stimulated with PPD. In some experiments IL-12 and anti-IL-10 were added to the cultures and IFN-γ was measured in supernatants.

The HTLV-1 proviral load was performed after DNA extraction from mononuclear cells as previously described [9].

### Statistical analysis

Hospitalized pulmonary TB patients with HTLV-1 were compared to control patients without HTLV-1 by Student *t*-test for continuous variables and Pearson *x*^2^ test for categorical variables. Fisher’s exact test was used when one the comparison groups had an expected frequency of five or less. Kaplan-Meier survival analysis was used to compare sputum microscopy conversion after 10 and 20 days of tuberculosis treatment. Comparison of TNF-α, IL-12 and IFN-γ production between the groups was performed by the Mann Whitney U Test. All analysis was performed with statistical software STATA 10.1 (Stata Corp, College Station, TX, USA).

## Competing interests

The authors declare that they have no competing interests.

## Authors’ contribution

MLB was responsible for the clinical evaluation of the patients, X-ray analysis, data analysis and preparation of the manuscript. OB, TB, IC, IB, FB and DP assisted MLB in the clinical evaluation and followed the patients during hospitalization. BF and LR performed the statistical analysis and review of the manuscript. SS, AS and EMC were responsible for immunological studies. MLB, EMC and LR also participated of the study design, data analysis and in the preparation of the manuscript. All authors read and approved the final manuscript.

## Pre-publication history

The pre-publication history for this paper can be accessed here:

http://www.biomedcentral.com/1471-2334/12/199/prepub
